# Imaging-Based Patterns of Failure following Re-Irradiation for Recurrent/Progressive High-Grade Glioma †

**DOI:** 10.3390/jpm13040685

**Published:** 2023-04-19

**Authors:** Debanjali Datta, Archya Dasgupta, Abhishek Chatterjee, Arpita Sahu, Kajari Bhattacharya, Lilawati Meena, Kishore Joshi, Ameya Puranik, Indraja Dev, Aliasgar Moiyadi, Prakash Shetty, Vikas Singh, Vijay Patil, Nandini Menon, Sridhar Epari, Ayushi Sahay, Tejpal Gupta

**Affiliations:** 1 Department of Radiation Oncology, Tata Memorial Centre, Mumbai 400012, Indiaarchya1010@gmail.com (A.D.);; 2 Homi Bhabha National Institute (HBNI), Mumbai 400012, India; 3 Department of Radio-Diagnosis, Tata Memorial Centre, Mumbai 400012, India; 4 Department of Medical Physics, Tata Memorial Centre, Mumbai 400012, India; 5 Department of Nuclear Medicine, Tata Memorial Centre, Mumbai 400012, India; 6 Department of Neurosurgery, Tata Memorial Centre, Mumbai 400012, India; 7 Department of Medical Oncology, Tata Memorial Centre, Mumbai 400012, India; 8 Department of Pathology, Tata Memorial Centre, Mumbai 400012, India

**Keywords:** high-grade glioma, re-irradiation, recurrence, MRI, PET, patterns of failure

## Abstract

Background: Re-irradiation (ReRT) is an effective treatment modality in appropriately selected patients with recurrent/progressive high-grade glioma (HGG). The literature is limited regarding recurrence patterns following ReRT, which was investigated in the current study. Methods: Patients with available radiation (RT) contours, dosimetry, and imaging-based evidence of recurrence were included in the retrospective study. All patients were treated with fractionated focal conformal RT. Recurrence was detected on imaging with magnetic resonance imaging (MRI) and/ or amino-acid positron emission tomography (PET), which was co-registered with the RT planning dataset. Failure patterns were classified as central, marginal, and distant if >80%, 20–80%, or <20% of the recurrence volumes were within 95% isodose lines, respectively. Results: Thirty-seven patients were included in the current analysis. A total of 92% of patients had undergone surgery before ReRT, and 84% received chemotherapy. The median time to recurrence was 9 months. Central, marginal, and distant failures were seen in 27 (73%), 4 (11%), and 6 (16%) patients, respectively. None of the patient-, disease-, or treatment-related factors were significantly different across different recurrence patterns. Conclusion: Failures are seen predominantly within the high-dose region following ReRT in recurrent/ progressive HGG.

## 1. Introduction

The standard of management in patients with diffuse high-grade gliomas (HGG), including glioblastoma (GBM), includes maximal safe resection followed by adjuvant radiation (RT) with concurrent temozolomide followed by adjuvant chemotherapy [1,2,3,4]. The recent classification of central nervous system (CNS) tumors by the World Health Organization (WHO) in 2021 has included histopathological and molecular evaluation for the integrated diagnosis of diffuse gliomas [5]. Following optimal treatment, in most GBM or HGG, the site of first recurrence is related to the index tumor bed or residual disease [6,7,8]. Re-irradiation (ReRT) is an effective modality for the treatment of recurrent/ progressive HGG, either as a single modality or in combination with re-excision and salvage chemotherapy [9]. Appropriate selection of patients is crucial in providing optimal benefits balancing the chances of improving survival with a higher risk of radiation necrosis following a second course of radiation. Patients with a shorter interval of disease recurrence, poor performance status, or disseminated disease are associated with poor prognosis, with the doubtful role of aggressive treatment regimens, including ReRT. Major deciding factors to consider in ReRT are time from the course of RT, tumor volume at recurrence, doses delivered to organs at risk, performance status, and molecular characteristics [10,11].

With the improvement of conformal radiation techniques, including intensity-modulated radiotherapy (IMRT), volumetric modulated arc therapy (VMAT), or proton beam therapy and image guidance, ReRT is increasingly practiced in the radiation oncology community [12]. In a well-selected cohort, the use of ReRT in recurrent/progressive HGG has resulted in improved patient-reported outcomes [13]. Reported progression-free and overall survival outcomes with ReRT are 9–12 months and 12–16 months, respectively [14]. In the recently reported NRG Oncology/ RTOG1205 trial, the median progression-free survival and overall survival were 7 months and 10 months for patients with recurrent GBM treated with a combination of Bevacizumab and ReRT [15]. However, there is a lack of consensus regarding the dose fractionation and delineation of target volumes for ReRT. Various dose-fractionation schedules have been reported for ReRT, including conventional fractionation, hypofractionated, or stereotactic radiosurgery [16]. 

Imaging has a critical role in contemporary oncology practice aiding in diagnosis, treatment planning, response evaluation, and surveillance following treatment completion [17]. Magnetic resonance imaging (MRI) is considered the gold standard for the management of brain tumors due to its better anatomical soft tissue clarity, multiparametric nature, as well as the availability of advanced functional sequences. Since the majority of ReRT practice entails conformal RT techniques, appropriate delineation of the target volume (TV) is highly critical to ensure the treatment of entire areas of recurrent or progressive disease. Simultaneously due consideration is to be given to the accurate localization of organs of risk (OAR) to avoid higher doses which can otherwise lead to unacceptably higher rates of toxicity due to cumulative doses from two courses of RT. The use of MRI is sine qua non for delineation of both TV and OAR, which is typically registered with radiation planning computed tomography images. Moreover, there is an emerging body of evidence suggesting the incremental role of positron emission tomography (PET) imaging in RT planning to identify areas of biologically active disease [18,19]. Similar to the lack of consensus regarding appropriate fractionation, the practice regarding volume delineation for gross disease and margins for the microscopic disease is variable in the ReRT setting [10,12]. Contouring enhancing volume on T1w MRI versus inclusion of both T1w enhancing volume and T2-FLAIR extension for volume delineation has been debated [10]. 

The literature regarding patterns of recurrence after ReRT for HGG is limited. Here, we present a well-selected cohort of 37 patients treated with conformal RT with target volumes delineated based on MRI. The subsequent areas of disease recurrence detected on MRI and PET imaging were mapped with previous target volumes (based on MRI) and dose distribution. 

## 2. Methods and Materials

### 2.1. Patient Selection

Adults (>18 years) with recurrent/progressive HGG treated with radical doses of ReRT (with/without concurrent or adjuvant chemotherapy) between January 2012 and June 2020 were considered in the retrospective study. Approval was obtained from the institutional ethics committee, and a waiver of consent was granted due to the retrospective nature of the study. All patients were required to have a histological diagnosis of diffuse HGG treated previously with radical doses of RT and documented progression on histology and/or imaging. The decision to treat with ReRT (along with other modalities including surgery and chemotherapy) was made after multidisciplinary discussion in the joint neuro-oncology meeting (JNOM) comprising radiation oncologists, neurosurgeons, neuroradiologists, nuclear medicine physicians, neuropathologists, medical oncologists, and pediatric oncologists. To be considered for ReRT, the time interval from the first course was typically 2 years (except for a few patients). Other factors regarding eligibility for ReRT included limited volume and localized recurrence, good performance status, and favorable molecular biology, e.g., IDH mutant gliomas or MGMT promoter methylation in GBM. Patients with available RT contours, dosimetry details, and imaging-based evidence of recurrence were included in the current analysis. Patients with inadequate follow-up details or absence of imaging during recurrence for co-registration were excluded. Furthermore, patients where RT details during the first course were not available or treated with palliative intent or hypofractionation during the first course, no definitive histological diagnosis, circumscribed gliomas, or pediatric age group were excluded.

### 2.2. Re-Irradiation Volume Delineation and Techniques

All patients were treated with a conformal technique with image guidance. MRI was considered part of RT planning for all patients. The gross tumor volume (GTV) included enhancing disease or cavity (in postoperative setting) delineated based on T1w contrast-enhanced MRI, and typically clinical target volume (CTV) had a 5–10 mm margin beyond the GTV, including the infiltrative disease. In general, areas of progressive T2-FLAIR signals suggestive of microscopic disease compared with previous MRIs were evaluated and considered in the CTV as appropriate. Whenever available, the PET imaging done during disease recurrence was used for target volume delineation to include all the areas of metabolically active disease. Organs at risk were contoured following a standard institutional practice. Planning target volume (PTV) included a margin of 3 mm beyond the CTV. All patients were treated with conventional fractionation 1.8 Gy–2.0 Gy/fraction with a total dose decided by the responsible radiation oncologist, with appropriate dose-volume constraints applied for critical OARs such as brainstem, optic nerves, and chiasma. All patients were treated with conventional linear accelerator (IMRT/VMAT) or helical tomotherapy with daily image guidance protocols. 

### 2.3. Adjuvant Treatment and Follow-Up Schedule

After completion of ReRT, the first follow-up imaging with MRI was scheduled for 1 month. The decision regarding adjuvant chemotherapy was based on the decision of JNOM and the evaluation of the patient and disease status and fitness to receive chemotherapy. As most patients were treated with chemotherapy, monthly visits were scheduled during treatment, and imaging with MRI was considered every 3–6 months. After completion of chemotherapy (or patients not treated with systemic therapy), clinical follow-up was scheduled every 3 months. Response/surveillance imaging was considered at the end of chemotherapy and, subsequently, every 6 months or as indicated clinically. The MRI sequences included T1w pre and post-contrast sequences, T2w propeller and FLAIR, MR spectroscopy, and diffusion-weighted imaging. Amino acid positron emission tomography (PET) imaging with Flouroethyl-L-Tyrosine (FET) or Fluorodopa (FDOPA) was undertaken in cases of equivocal findings on MRI.

### 2.4. Analysis of Failure Patterns

Recurrence was documented as per decision in the tumor board based on imaging with MRI and/or PET. Recurrence on MRI was defined using Response Assessment in Neuro-oncology (RANO) criteria. On FET PET, recurrence was documented when the maximal standardized uptake value within the tumor was corrected for nonspecific uptake in the background (SUVmax/BG > 2.65). For FDOPA, recurrence was considered when the radiotracer uptake in the concerned region of interest was higher compared to substantia nigra. 

The patterns of failure were determined after rigid registration of the planning CT with MRI or PET (documenting recurrence). Recurrence volumes were contoured individually by a radiation oncologist, in concurrence with a neuroradiologist, both blinded to previous target volumes and isodose lines. Recurrence patterns were classified based on the volume of recurrent disease within the 95% isodose line: central (>80% volume), marginal (20–80% volume), and distant (<20% volume). 

### 2.5. Statistical Analysis

All the patient, disease, and treatment-related characteristics were obtained from retrospective review of electric medical records, radiation charts, and radiation plans. Demographical and clinicopathological variables were summarized using descriptive statistics and compared across recurrence patterns using the Pearson chi-square test and Fisher’s exact test as appropriate. Survival analysis was performed using the Kaplan–Meier product limit method, using log-rank test for univariate analysis. For survival estimation following ReRT, the date of starting ReRT was considered as the baseline. The radiological evidence of disease recurrence was considered for analysis of progression-free survival (PFS), and the date of death from any cause was considered for analysis of the overall survival (OS). Any *p*-value of <0.05 was considered statistically significant. All the statistical analyses were performed using the Statistical Package for the Social Sciences (SPSS version 24; IBM Corp., Armonk, NY, USA).

## 3. Results

Thirty-seven patients with recurrent/ progressive HGG were included for analysis. The clinical and treatment characteristics during the initial diagnosis are summarized in Table 1. Most patients were diagnosed with astrocytoma (n = 35), with grade 3 histology described in 23 patients. The median dose during the first course of RT was 56 Gy (range 54–60 Gy). Regarding the RT technique, 15 patients received the 2D technique, while the remaining received 3D-conformal (n = 30) or IMRT (n = 2). Chemotherapy was received by the majority of patients, concurrent in 23 and adjuvant in 21. Molecular information with IDH mutation was available in 24 of 37 patients (either during presentation or recurrence), with 15 (63%) patients having IDH mutant glioma. Oligodendroglioma with 1p19q codeletion was seen in two patients. During diagnosis, 14 patients had grade 4 astrocytoma, of which MGMT gene promoter was methylated in 5 patients out of 8 where it was available.

The details of treatment during recurrent disease are noted in Table 2. The time interval from the first course of RT to ReRT was 44 months (range 18–169 months). The interval between two RT courses was <2 years in 1 patient only (18 months), while in 12 patients, it was >5 years. The majority of patients were considered for surgery (n = 34) before ReRT, with most patients undergoing subtotal resection (n = 22) followed by gross total resection (n = 9) and biopsy (n = 3). Nine patients had received chemotherapy before consideration of ReRT. The median dose of ReRT was 54 Gy (range 45–59.4 Gy). The median cumulative EQD2 from two courses of RT was 106.2 (range 96.2–113.3). Radiation was delivered using helical tomotherapy in 22, while IMRT/VMAT on a linear accelerator was used in 15. Concurrent chemotherapy with temozolomide was given to 25 patients. Adjuvant chemotherapy following ReRT was used in 29 patients, with the median number of cycles being 6 (range 2–18). 

**Figure 1:**
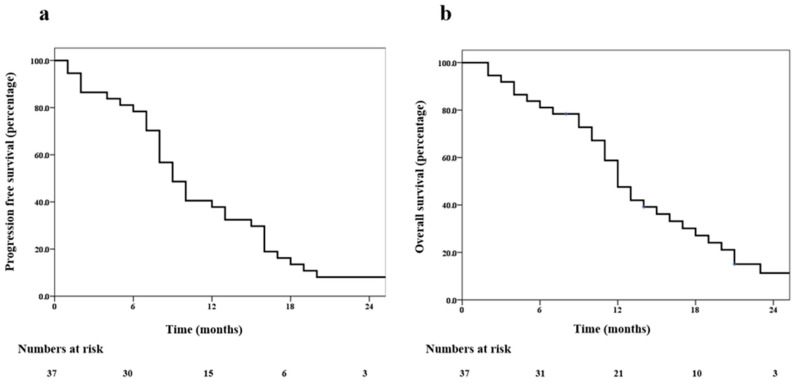
Progression-free survival (**a**) and overall survival (**b**) following re-irradiation in high-grade glioma.

**Figure 2:**
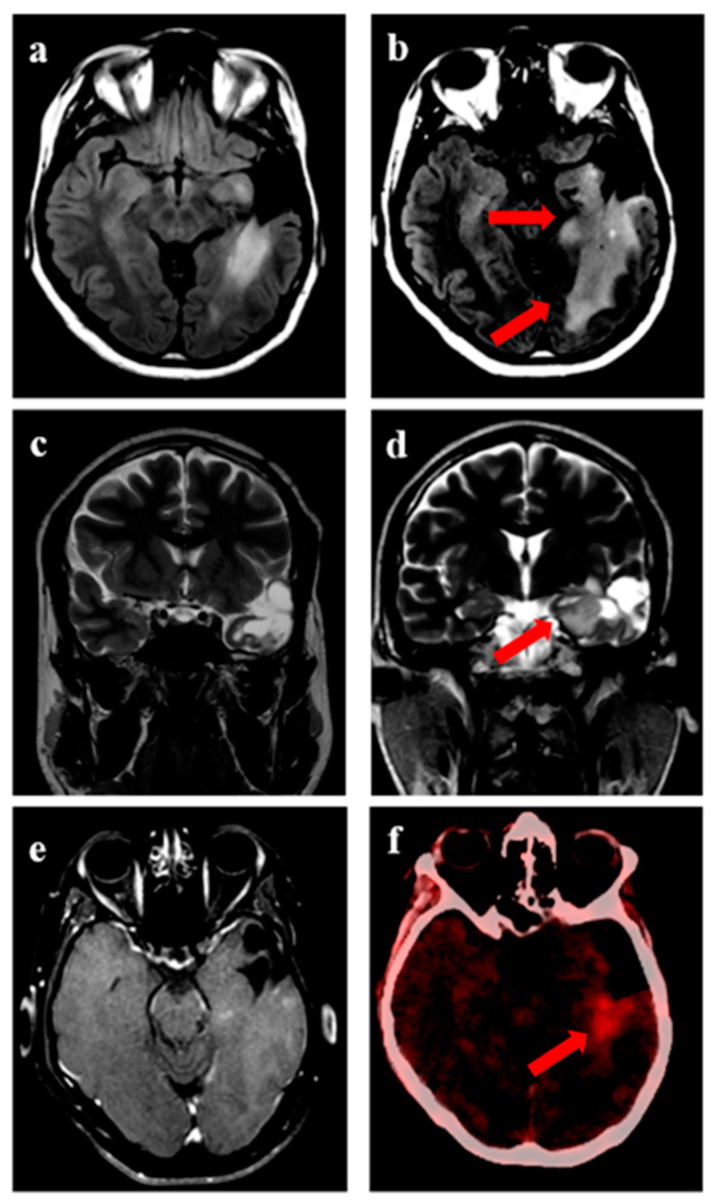
Progression in a 32-year male with IDH-mutant astrocytoma. (**a**) shows residual disease following treatment completion, with (**b**) showing an increase in disease extent (arrow) after 7 years, as appreciated on axial T2-FLAIR sequences when reirradiation was considered. Representative images showing an increase in disease extent over medial extent of the cavity on coronal T2w sequence (**c**,**d**). (**e**) shows T1w-post gadolinium images showing no uptake of contrast in the area of new disease, suggesting absence of transformation to grade 4. (**f**) shows PET avidity over the area of active disease.

**Figure 3:**
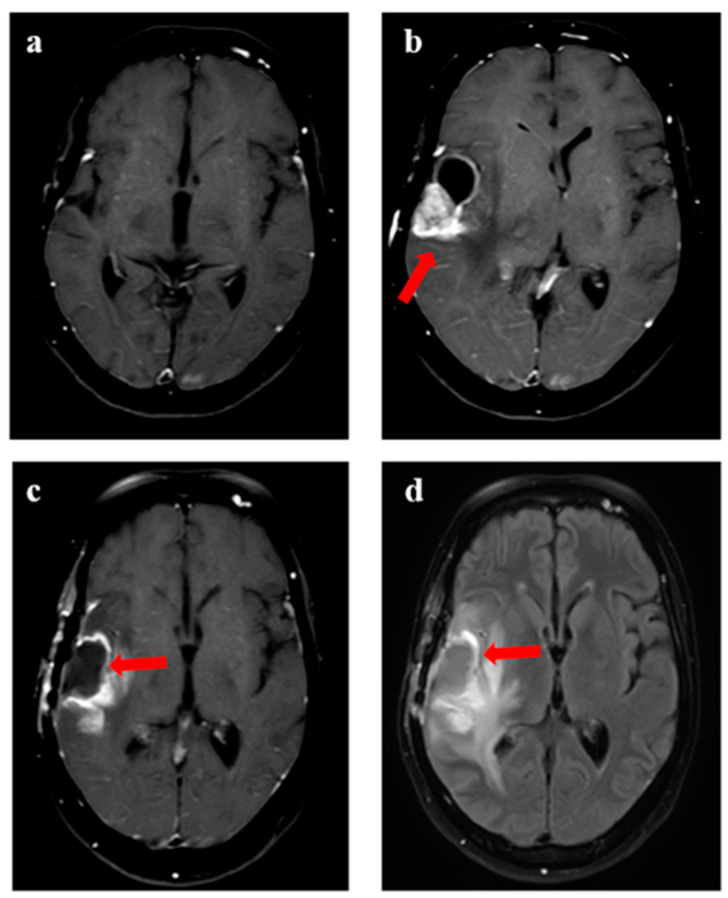
Progression in a 47-year female with glioblastoma with MGMT gene promoter methylation. (**a**) shows T1w axial view following completion of adjuvant chemotherapy (after surgery and radiation), with (**b**) showing local recurrence after 2.5 years with enhancing component and cystic component (arrow). (**c**,**d**) represented the surgical cavity on T1w contrast and T2w FLAIR axial view when the patient was considered for reirradiation.

**Figure 4:**
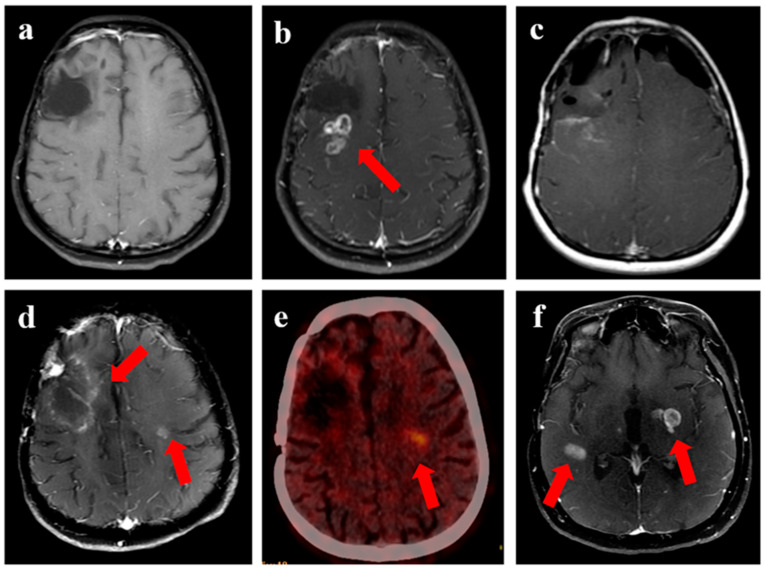
Disease evolution from first progression to subsequent recurrence following re-irradiation in a 25-year female with IDH-mutant grade 3 astrocytoma. (**a**) shows T1w contrast-enhanced MRI with resection cavity over the right frontal region, with contrast-enhancing recurrent disease seen over the posterior aspect of the cavity (arrow) in (**b**). Subsequent imaging shows MRI following resection of the disease in (**c**), which was confirmed as recurrent HGG. (**d**) shows an enhancing area with central necrosis over the resected and re-irradiated region (8 months following ReRT) over the right frontal-parietal, which was not showing any metabolic uptake on corresponding PET imaging in (**e**). However, the other area of enhancing nodule over the left periventricular region shows high avidity, considered a distant recurrence. (**f**) shows MRI performed after 2 months, which confirmed new areas of distant disease, while the re-irradiated region (non-avid on PET) continued to be stable, confirming RT-related changes.

The median time to recurrence was 9 months (range 1–47 months). The progression-free survival at 6 and 12 months was 78% (95% confidence interval (CI) 64–90%) and 38% (95% CI 23–62%), respectively (Figure 1a). The overall survival at 6 months and 12 months was 81% (95% CI 69–94%) and 59% (95% CI 42–74%), respectively (Figure 1b). Representative images for one patient with grade 3 glioma and one patient with GBM are shown in Figure 2 and Figure 3, respectively. During recurrence, the available imaging modality was MRI in 27, PET in 3, and both MRI and PET in 7. Figure 4 shows a patient undergoing surgery followed by radiation (Figure 4c), (postoperative pre-ReRT scan) and ReRT with disease recurrence on MRI and PET. The index area of recurrence (resected and irradiated) over the right frontal-parietal region showed increased contrast-enhancement (4d), which remained non-avid on PET (Figure 4e) (considered radionecrosis), while other enhancing areas over the contralateral periventricular region were showing metabolically active disease (Figure 4e) (distant recurrence). Follow-up scans confirmed the re-irradiated region to represent radionecrosis as it remained stable while the distant disease progressed further (Figure 4f). There was no significant difference in 1-year PFS for patients with or without IDH mutation (30% vs. 32%, respectively, *p* = 0.85). Given a small number of patients with 1p19q codeletion or known MGMT gene promoter methylation, no separate analysis was undertaken for these groups. The PFS was trending towards significance, favoring better outcomes for patients with gross total resection or subtotal resection compared to biopsy or no surgery, with a 1-year PFS of 40% (95% CI 33–50%) vs. 17% (95% CI 2–32%), respectively (*p* = 0.06). 

**Figure 5:**
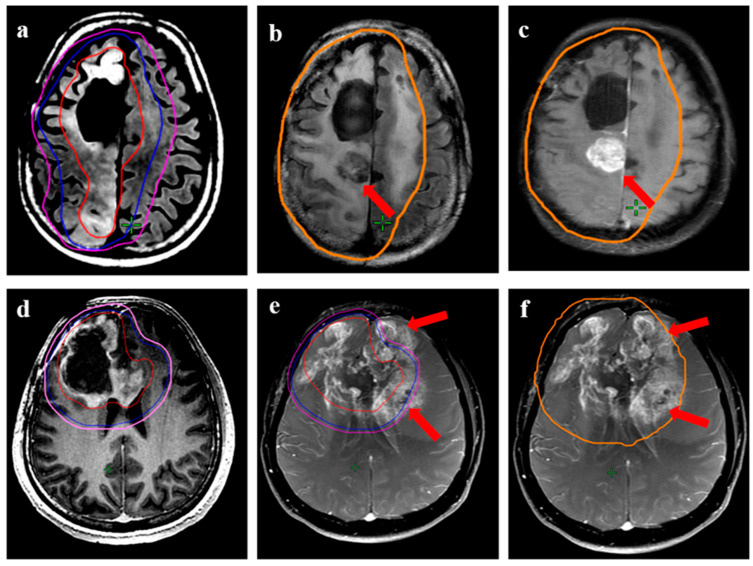
Two patients with central recurrence, each in the upper and lower panel. (**a**) (37-year male with IDH-mutant astrocytoma) shows target volumes (GTV: red, CTV: blue; PTV: magenta) on T2-FLAIR MRI. The CTV was drawn to include all areas of T2w hyperintensity. (**b**,**c**) is T2-FLAIR and T1w contrast images showing recurrent disease (arrow) and its relation with the 95% isodose line (orange line). (**d**) (56-year male with recurrent glioblastoma) shows the target volumes for another patient volumes (GTV: red, CTV: blue; PTV: magenta) on T1w contrast MRI. The GTV was the enhancing disease, with CTV drawn as an expansion of 10 mm around the enhancing disease. (**e**,**f**) show the recurrence volumes with respect to target volumes and the 95% isodose line (orange), respectively. The major portion of recurrent disease can be seen lying within the 95% isodose line.

**Figure 6:**
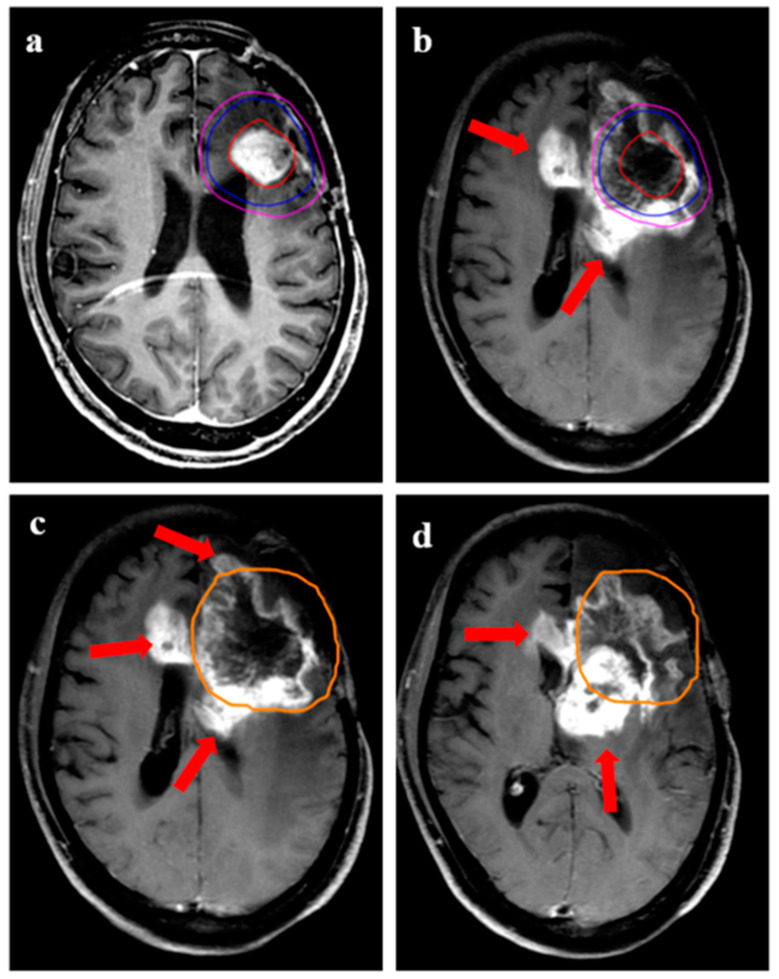
Demonstration of marginal recurrence following re-irradiation for a 43-year female with transformed high-grade glioma. (**a**) shows the target volumes (GTV: red, CTV: blue; PTV: magenta) on T1w contrast MRI. The GTV was the enhancing disease, with CTV drawn as an expansion of 10 mm around the enhancing disease. (**b**,**c**) demonstrates recurrent disease (arrows) with respect to target volumes and 95% isodose line (orange), 1. (**d**) is a representation from lower axial slices showing the growth of recurrent disease further beyond the 95% isodose line.

**Figure 7:**
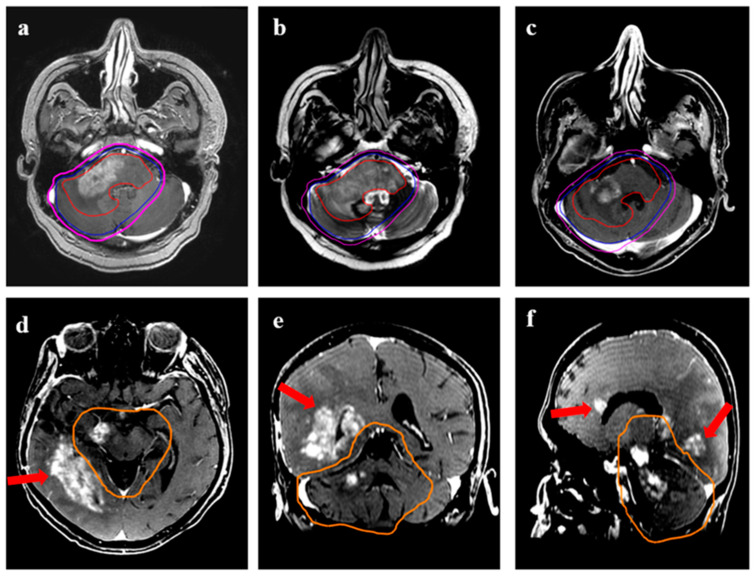
Distant relapse following re-irradiation in a 38-year male with IDH-mutant astrocytoma with transformation (radiological) to grade 4 during reirradiation. (**a**,**b**) show the target volumes (GTV: red, CTV: blue; PTV: magenta) on T1w contrast and T2w images. The GTV encompassed all the T2w altered signals, including the enhancing disease, while CTV was 10–15 mm expansion along the brain parenchyma. (**c**) represents the response in the primary re-irradiated region 6 months following ReRT, while the lower panel shows axial (**d**), coronal (**e**), and sagittal (**f**) T1w contrast MRI from the same time-point demonstrating distant areas of recurrent disease (arrow) beyond the 95% isodose line (orange).

Central, marginal, and distant recurrences were seen in 27 (73%), 4 (11%), and 6 (16%), respectively (Table 3). Of 27 patients with central or in-field recurrence, 14 had more than 50% of the recurrent disease volume within GTV. In the remaining 13 patients, the predominant area of recurrent disease (>50% volume) was seen in the region between GTV and CTV. Figure 5 demonstrates two patients having a central recurrence. In the upper panel, a patient developed recurrence (Figure 5c) within the GTV (contoured based on T2-FLAIR hyperintensity). In the lower panel, the patient developed recurrence primarily beyond the GTV; however, it encompassed the 95% isodose line (Figure 5e,f). Representative images are shown for marginal recurrence (Figure 6) and distant recurrence (Figure 7). Out of six patients with distant disease recurrence in the brain parenchyma, three patients were seen to have leptomeningeal disease (LMD) of disease in the brain. For patients with non-central recurrence, IDH status was known for seven patients, five of whom were mutant. 

The time to recurrence following ReRT between the three recurrent patterns was not significantly different, with the median time for central, marginal, and distant recurrences being 10 months, 11 months, and 8 months, respectively. Furthermore, a separate analysis was performed to study if clinical or treatment factors were different for central vs. non-central (marginal and distant combined), but none of the factors were seen to be statistically significant. However, the longer time to ReRT (>4 years) was seen in 8 of 10 patients for non-central versus 12 of 27 patients with central recurrence (*p* = 0.053). Chemotherapy (concurrent and/or adjuvant) was used in 24 of 27 patients with central recurrence compared to 7 of 10 patients with non-central relapse (*p* = 0.19). 

## 4. Discussion

Re-irradiation constitutes an effective treatment modality for appropriately selected recurrent/progressive HGG. The practice of RT target delineation and dose-fractionation is widely variable in re-irradiation settings, with a paucity of literature on the influence of such parameters on recurrence patterns. The current study describes recurrence patterns in a cohort of patients uniformly treated with radical doses of RT using MRI-based target volumes. 

In patients with GBM or HGG, the majority (80–90%) of recurrences had been reported to be in relation to the index tumor bed and high-dose region [7,8]. Although the predominant recurrence pattern following ReRT reported in the literature continues to be in the high-dose region or in-field, the proportion appears relatively lesser in the range of 50–70% compared to de novo HGG. This can be reasoned by conservative margins used during ReRT due to concerns of higher incidence of radionecrosis increasing the likelihood of marginal recurrence occurring in proximity to the high-dose region. Upon recurrence, gliomas had been demonstrated to be genetically diverse, with multiple acquired molecular pathways depicting aggressive biological behavior and outcomes, which could explain the higher incidence of distant relapses [20,21]. 

Our study reports in-field failure in 73% of patients, with 11% having marginal and 16 % with distant failure. Shapiro et al. analyzed failure patterns in recurrent glioma patients after treatment with ReRT and bevacizumab [22]. Hypofractionated stereotactic RT to a dose of 30 Gy in five fractions was used. The predominant pattern of failure was in-field (53%), 24% had marginal failure, and 24% had an out-of-field failure. The higher rates of marginal relapse may indicate the rapid-dose fall with radiosurgery treatment. Niyazi et al. reported 61% in-field failure, 23% marginal failure, and 16% out-of-field failure with conventionally fractionated ReRT dosage of 36 Gy in 18 fractions with concurrent bevacizumab [23]. The published literature has demonstrated that a well-selected cohort of patients like recurrent oligodendroglioma, IDH mutant astrocytoma, and MGMT gene promoter methylated GBM have better outcomes following salvage reirradiation [14]. In the current study, the major factor in deciding on ReRT was a longer interval to recurrence following the first course of radiation for patients with grade 4 gliomas selecting the better cohort of patients. Additionally, it is important to consider the median interval between two courses of RT in our group was 44 months, which is lower than the median PFS for IDH mutant gliomas, suggesting a group of patients having bad biology with recurrence earlier than expected. This can be a contributory factor for no observed difference in PFS in our cohort based on IDH mutation status. In the literature, the doses of radiation used in the setting of ReRT are widely variable. For recurrent GBM, many groups prefer using a hypofractionated regimen (including the recently reported RTOG 1205) with typical median survival rates of less than 12 months [15]. In our cohort, we have used normofractionated radiation to higher biological doses given a longer interval between two courses of RT (median 44 months) with a 1-year OS of 59%. The role of systemic therapy in treating recurrent gliomas includes using agents such as bevacizumab, temozolomide, and lomustine [2]. The choice of systemic agents and timing or sequencing (when surgery or radiation is considered) for recurrent gliomas is widely variable. Systemic therapy should be considered in patients with longer intervals to recurrence after initial treatment, in chemotherapy-naïve patients, and particularly for IDH-mutant gliomas, including oligodendroglial histology. In our study, the most frequent chemotherapy used was temozolomide following ReRT.

In the ReRT setting, there is a lack of consensus regarding volume delineation. The practice can vary from the treatment of T1w contrast-enhancing volume only versus the inclusion of FLAIR signal abnormalities [10]. While target delineation, it is crucial to carefully evaluate the temporal changes in the concerned MRI and account for index histology since recurrence can represent an increase in disease extent (for IDH-mutant gliomas) or transformation to higher grades. Multiparametric MRI with functional sequences should be considered whenever feasible. In cases of an increase in the volume of non-enhancing T2w hyperintensity (progression of extent) without definitive areas of contrast uptake, it will be essential to consider all the T2w abnormalities in the initial GTV. Similarly, for gliomas suggesting transformation within the previous extent of the residual tumor as indicated by new areas of contrast enhancement, areas of hyperperfusion, or higher choline peaks, it will be prudent to consider all the areas of residual tumor and new-enhancing tumor in the target volumes. However, for IDH-wild GBM with clear progression as T1c enhancing lesion, it might be reasonable to treat the enhancing disease with limited margins. It is also important to consider there can be uncertainties in the peritumoral region (PTR) of GBM, with both microscopic disease or vasogenic edema appearing similar on conventional MRI sequences. Quantitative image analysis has shown promise in identifying microscopic infiltrative tumors within the PTR [24,25]. From our study findings, contouring with the use of MRI and including T1w enhancement for well-defined enhancing recurrence with limited CTV margins or T2w hyperintense areas for grade 3/transformed grade 4 tumors, and the use of smaller PTV margins with image guidance did not lead to an increased marginal miss. Although the median PTV volume of 290 cc in our study was relatively larger, conventional fractionation resulted in good compliance with ReRT. 

The role of PET imaging is increasingly recognized in neuro-oncology [19]. The use of amino acid PET can be considered in target volume delineation, as it can aid in identifying areas of infiltrative disease that can otherwise remain radiologically silent on conventional MRI sequences. Furthermore, areas of the tumor with a higher degree of radiotracer uptake can potentially represent areas of higher-grade or resistant clones, opening the window for radiation dose painting or adaptive RT to deliver higher doses to improve control rates. Laack et al. reported the use of FDOPA PET-guided dose escalation in newly diagnosed GBM with promising results improving survival in MGMT methylated patients [26]. The use of PET in the planning of ReRT is worth exploring in future trials and is expected to help target delineation and identify areas of biologically active disease. Moreover, given that these patients have been treated with RT earlier, some of the MRI findings can represent radiation-induced changes that can be identified with metabolic imaging. The use of biological imaging to delineate between recurrence and treatment-induced changes has shown PET has an excellent diagnostic accuracy; 89% positive predictive value [27] and thereby may aid in cases with equivocal MRI findings. In our study, 20% of patients had both MRI and PET for diagnosing recurrence following ReRT. The role of advanced imaging with different radioisotopes and functional MRI sequences in newly diagnosed and recurrent gliomas will contribute to a better diagnosis of intratumoral heterogeneity and treatment planning, paving the way toward personalized radiation planning. For patients with IDH-mutant lower glade gliomas, it is well-known areas of higher grade can be present, which can remain undiagnosed on histopathological evaluation alone due to the absence of tissue when subtotal resection is conducted. Advanced imaging can contribute significantly to such scenarios for noninvasive identification of the differential characteristics within the tumor region. Moreover, PET or advanced MRI can be used to demarcate areas of infiltrative tumor from vasogenic edema in the peritumoral region of GBM, which can aid in planning personalized treatment. Differential radiation dose prescriptions in the form of dose painting or adaptive radiation can be explored guided by biological imaging like PET and advanced MRI sequences such as spectroscopy for optimizing disease control and toxicity.

Reported toxicity after ReRT varies, with 5–20% of patients experiencing radionecrosis [28,29]. We hereby have not analyzed the toxicity rate; however, previously published literature on 111 patients from the same institute treated with conventional fractionation ReRT for HGG had reported 12% of patients developing frank radionecrosis [14]. The use of antiangiogenic agents such as bevacizumab used along with ReRT can possibly lead to reduced rates of radionecrosis and has the potential to improve survival as well from antitumoral activity [29,30], which is being planned to be investigated in a phase III randomized controlled trial at our institute [31]. 

The study has the intrinsic limitations of a retrospective study. Molecular markers, an important prognostic and predictive factor for gliomas, were unavailable for a proportion of patients. Since a proportion of patients received RT elsewhere and were also treated using 2D techniques, cumulative dosimetric analysis was not possible for this study. Furthermore, during recurrence, the use of chemotherapy (concurrent and maintenance) was variable, which can have possible implications on the clinical outcomes. Since IDH mutation status was not available in a proportion of patients, a precise classification of all tumors according to WHO 2021 classification was not possible.

A higher incidence of in-field recurrence with conformal ReRT implies there is possible scope for improvement of control rates with higher doses of RT. Future studies of dose-escalation with simultaneous integrated boost guided by functional MRI and PET for dominant/ metabolically active disease are warranted to improve local control, particularly in patients with longer intervals from the first course of RT. In the future, with a higher number of patients treated with ReRT, contouring recommendations can be established for recurrent GBM, transformed IDH-mutant gliomas, or progressive IDH-mutant gliomas (without transformation to higher grade) individually. 

## 5. Conclusions

The use of MRI-guided target volume delineation to deliver radical doses of focal ReRT using conventional fractionation leads to acceptable control rates in recurrent/progressive HGG. The predominant recurrence pattern is central (within the high-dose region) in 73%, while marginal and distant relapses are seen in 11% and 16% of patients, respectively. 

## Figures and Tables

**Table 1:** Clinical characteristics and details of first course of treatment.

Features	Frequency
Gender	
Male	28 (76%)
Female	9 (24%)
Grade	
Grade 3	23 (62%)
Grade 4	14 (38%)
Histology	
Astrocytoma	35 (95%)
Oligodendroglioma	2 (5%)
IDH ^a^	
Mutant	15 (41%)
Negative	9 (24%)
Non-contributory/ Unknown	13 (35%)
ATRX ^a^	
Lost	16 (43%)
Retained	7 (19%)
Non-contributory	14 (38%)
Dose: first course of radiation	
Median (range)	56 (54–60) Gy
Technique	
2D-conventional	15 (41%)
3D-conformal	20 (54%)
IMRT	2 (5%)
Concurrent chemotherapy	
Yes	23 (62%)
No	14 (38%)
Adjuvant chemotherapy	
Yes	21 (57%)
No	16 (43%)

IMRT: Intensity-modulated radiotherapy. ^a^ Includes molecular information when available following recurrence (when not available during first diagnosis).

**Table 2:** Clinical characteristics during recurrence and details of treatment.

Features	Frequency
Age (during re-irradiation)	
Median (range)	40 (24–60) years
Time from first radiation	
Median (range)	44 (18–169) months
Surgery	
No surgery	3 (8%)
Biopsy	3 (8%)
Subtotal resection	22 (59%)
Gross total resection	9 (24%)
Grade during recurrence ^a^	
Grade 3	4 (12%)
Grade 4	33 (88%)
Performance status during re-irradiation	
KPS ≥ 80	21 (57%)
KPS 50–70	16 (43%)
Dose during reirradiation	
Median (range)	54 (45–59.4) Gy
Cumulative EQD2	
Median (range)	106.2 (96.2–113.3)
PTV volume during re-irradiation	
Median (range)	290 (75–704) cc
Concurrent chemotherapy during re-irradiation	
Yes	25 (68%)
No	12 (32%)
Chemotherapy	
Concurrent chemotherapy alone during re-irradiation	2 (5%)
After reirradiation (with or without concurrent)	29 (78%)
No chemotherapy	6 (16%)

KPS: Karnofsky Performance Status; EQD2: Equivalent dose in 2 Gy per fraction (considering α/β of 10). ^a^ For patients who didn’t undergo surgery (n = 3), radiological features during recurrence were taken into consideration.

**Table 3:** Features during recurrence following re-irradiation.

Features	Frequency
Imaging modality during recurrence	
MRI alone	27 (73%)
PET alone	3 (8%)
MRI and PET	7 (19%)
Time to recurrence after re-irradiation	
Median (range)	9 (1–47) months
Volume of recurrence following re-irradiation	
Median (range)	69.5 (3.3–221) cc
Patterns of recurrence (with respect to 95% isodose)	
Central	27 (73%)
Marginal	4 (11%)
Distant	6 (16%)
Time to recurrence (for different patterns)	
Central	
Median (range)	10 (1–47) months
Marginal	
Median (range)	11 (6–16) months
Distant	
Median (range)	8 (2–19) months

MRI: Magnetic resonance imaging; PET: Positron emission tomography.

## Data Availability

Data can be shared on reasonable request to the corresponding author following institutional ethics committee guidelines.
